# Optimization of Ultrasonicated Kaempferol Extraction from *Ocimum basilicum* Using a Box–Behnken Design and Its Densitometric Validation

**DOI:** 10.3390/foods9101379

**Published:** 2020-09-29

**Authors:** Ammar B. Altemimi, Muthanna J. Mohammed, Lee Yi-Chen, Dennis G. Watson, Naoufal Lakhssassi, Francesco Cacciola, Salam A. Ibrahim

**Affiliations:** 1 Department of Food Science, College of Agriculture, University of Basrah, Basrah 61004, Iraq; 2 Department of Biology, College of Education for Pure Sciences, University of Mosul, Mosul 41002, Iraq; mjmk73@yahoo.com; 3 School of Agricultural Sciences, Southern Illinois University at Carbondale, Carbondale, IL 62901, USA; yclee010689@gmail.com (L.Y.-C.); dwatson@siu.edu (D.G.W.); naoufal.lakhssassi@siu.edu (N.L.); 4 Department of Biomedical and Dental Sciences and Morphofunctional Imaging, University of Messina, 98125 Messina, Italy; 5 Food and Nutritional Sciences Program, North Carolina A & T State University, Greensboro, NC 27411, USA; ibrah001@ncat.edu

**Keywords:** kaempferol, *Ocimum basilicum*, high performance thin layer chromatography in connection with ultraviolet detection (HPTLC-VIS), box-Behnken design, optimization, validation

## Abstract

Kaempferol (KA) is a natural flavonol that can be found in plants and plant-derived foods with a plethora of different pharmacological properties. In the current study, we developed an efficient extraction method for the isolation of KA from ultrasonicated basil leaves (*Ocimum basilicum*). We successfully employed a Box–Behnken design (BBD) in order to investigate the effect of different extraction variables including methanol concentration (40–80%), extraction temperature (40–60 °C), and extraction time (5–15 min). The quantification of KA yield was carried out by employing a validated densitometric high performance thin layer chromatography in connection with ultraviolet detection (HPTLC-VIS). The obtained data showed that the quadratic polynomial model (R^2^ = 0.98) was the most appropriate. The optimized ultrasonic extraction yielded 94.7 ng/spot of KA when using methanol (79.99%) at 60 °C for 5 min. When using toluene-ethyl acetate-formic acid (70:30:1 *v*/*v*/*v*) as a solvent, KA was detected in basil leaves at an Retention factor (Rf) value of 0.26 at 330 nm. Notably, the analytical method was successfully validated with a linear regression of R^2^ = 0.99, which reflected a good linear relationship. The developed HPTLC-VIS method in this study was precise, accurate, and robust due to the lower obtained results from both the percent relative standard deviation (%RSD) and SEM of the *O. basilicum.* The antioxidant activity of KA (half maximal inhibitory concentration (IC_50_) = 0.68 μg/mL) was higher than that of the reference ascorbic acid (IC_50_ = 0.79 μg/mL) and butylated hydroxytoluene (BHT) (IC_50_ = 0.88 μg/mL). The development of economical and efficient techniques is very important for the extraction and quantification of important pharmaceutical compounds such as KA.

## 1. Introduction

Plants are sources of various phytochemicals such as terpenes, phenylpropanoids, diarylheptanoids, isothiocyanates, and sulfur compounds. These natural compounds are often explored for their potential use. For example, phytochemicals produced by herbs and spices are of interest due to their culinary and medicinal uses. Some of the phytochemical functions include antioxidant activities, the modulation of detoxification enzymes, the enhancement of the immune system, the reduction of inflammation, the modulation of steroid metabolism, antiviral and antibacterial effects, and the oxidative retardation of lipids [1,2]. Medicinal herbs can also be an alternative source of antioxidants outside of vitamin C, vitamin E, and carotenoids [3].

Basil (*Ocimum basilicum* L.) is an herbaceous annual plant that generally produces white-purple flowers and is one of the most important cultivated aromatic herbs in the world. The *Ocimum* genus belongs to the mint family (*Lamiaceae*), which contains dozens of medicinal plants that are grown for their high economic value. This genus is native to tropical and subtropical regions of Asia, Africa, and South America, and it consists of annual and perennial herbs and shrubs [4,5]. Basil can be cultivated in fields as well as under greenhouse conditions, which can result in different concentrations of chlorophylls and secondary metabolites. This herb is utilized for its medicinal values to treat headaches, coughs, diarrhea, constipation, warts, worms, and kidney malfunction [6]. Moreover, basil is an essential ingredient used in traditional culinary practices [7,8] and is known as rihan in Arabic [9]. Basil has many health benefits, and it is composed of essential nutrients such as vitamin A, vitamin C, calcium, phosphorus, and beta carotene [10]. It also contains flavonoids and phenolic compounds that act as reducing agents that contribute to its antioxidant activities [11]. Basil extract is commonly used as an herbal drug due to the many pharmacological effects such as anti-hyperglycemic [12], hypolipidemic, antiatherosclerotic [13], and anticancer activities [14]. Basil leaves contain several polyphenols, bioactive compounds [15], and phenolic acids (caffeic acid, caftaric acid, and rosmarinic acid) [16], and they are also rich in flavonoids such as anthocyanins, quercetin, kaempferol (KA), and luteolin. A very recent study conducted on the natural populations of basil indicated that it may have different chemical composition and biological activities [17]. Among flavonoids, KA is a natural flavonol antioxidant found in many fruits and vegetables. Several studies have focused on dietary KA due to its health benefits. In particular, it has been shown that KA may lower the risk of some chronic diseases, especially cancer [18]. In addition, KA has been reported to increase the body’s antioxidant defense against free radicals, the common cause of cancer, resulting in an inverse relationship between the use of dietary KA and cancer [19,20]. Due to the high pharmacological benefits of basil, researchers worldwide are currently interested in developing simple, efficient, and reliable techniques to analyze different plant extracts for pharmaceutical purposes.

Plant extracts can be analyzed with Thin-Layer Chromatography (TLC) [21], Gas Chromatography-Mass Spectrometry (GC-MS) [22], and High-Performance Liquid Chromatography (HPLC) [23,24]. In recent years, the high performance TLC (HPTLC) densitometry method has grown in popularity because it is economic, sensitive, accurate, and reduces the time needed to process large samples. The increased sensitivity of this method and the ability to process many samples in short periods of time have led researchers to use TLC-densitometry over the popular HPLC [25]. Therefore, the aim of the present work was to optimize the ultrasonic-assisted extraction of KA from *Ocimum basilicum* by applying a Box–Behnken design (BBD) and to quantify such a flavonol with a simple, efficient, and validated HPTLC-VIS technique. 

## 2. Materials and Methods 

### 2.1. Sample and Sample Preparation

Samples of basil (*Ocimum basilicum*) were collected in Basrah, Iraq, and authenticated by the horticulture faculty at the College of Agriculture University of Basrah, Basrah, Iraq. Basil leaves were washed with distilled water, crushed in a blender, and then sealed in plastic bags and stored at −18 °C for two days before freeze-drying. Samples were shielded from light.

### 2.2. Solvent Mixture Screening

Different solvent mixtures (Table 1) were tested in order to maximize the extraction of total flavonoids. The higher flavonoid content positively reflected the amount of the desired KA compound. For each of the five solvent mixtures, 2 g of lyophilized basil were ground with a mortar and pestle with 20 mL of the appropriate solvent mixture. The mixture was transferred to a flask, and 100 mL of a different solvent mixture was added. The resulting solution was then transferred to a 200 mL cylindrical polypropylene container with a screw-on lid before insertion into an ultrasonic bath cleaner (UBC; Elmasonic P30, Elma Hans Schmidbauer GMBH, Singen, Germany) at a 37 kHz frequency, 35 W/cm^2^ power, and 60 °C for 15 min.

### 2.3. Total Flavonoid Content 

The total flavonoid content of each sample was quantified with the aluminum chloride colorimetric method [26]. Quercetin was used to make the standard calibration curve for total flavonoid content. Quercetin (5 mg) was dissolved in 1 mL of methanol, after which serial dilutions were prepared with methanol (5–200 mg/mL). Each of the serial dilutions was mixed with 0.6 mL of 2% aluminum chloride and incubated at room temperature for 60 min. A UV–Vis spectrophotometer (Sunny UV.7804C, Tokyo, Japan) was used to measure the absorbance of the reaction mixtures at the 420 nm wavelength and compared to a blank slide.

### 2.4. Ultrasonic-Assisted Extraction of KA

Lyophilized *Ocimum basilicum* (10 g) was weighed and placed in each one of the 200 mL glass flasks used. Different percentages of methanol (40%, 60%, and 80%) with 100 mL of solvent each were added to corresponding flasks and moved to a 120 mL cylindrical polypropylene container with a screw-on lid before insertion into the UBC. The UBC was operated at a 37 kHz frequency and 50% constant power for all treatments, and an adjustable water bath was used to reach the desired temperature before initiating ultrasonic treatment. According to the manufacturer’s effective power rating, an ultrasonic power at a 50% power setting was used at 35 W/cm^2^. The selected mixture was investigated at three different temperatures (40, 50, and 60 °C) over three ultrasonic treatment periods of 5, 10, and 15 min. Each UBC setting was repeated in triplicate with samples before changing to the next setting. After UBC treatment, the upper layer of samples was filtered (Whatman no. 1 paper) and subjected to rotary evaporation at 40 °C with a vacuum in order to remove the solvent [1].

### 2.5. Column Chromatography 

Sequential purification through column chromatography was employed for partial purification for 14 different experiments with ultrasonicated *Ocimum basilicum* extracts. For the stationary phase, activated silica gel (pore size 60–120 mesh) was used, and for the mobile phase, a sequence of n-hexane, ethyl acetate, and petroleum ether was used. Additional column chromatography was completed after the crude methanol residue was partially dissolved in n-hexane and triturated with silica. Fractions were collected starting with n-hexane followed by ethyl acetate and petroleum ether. The collected fractions were then condensed. Due to their high fatty acid content, n-hexane and petroleum ether were discarded. A rotary evaporator was used to dry the collected ethyl acetate fractions before weighing. The result was yellow crystals of ethyl acetate fractions in test tubes. Each fraction was tested in order to confirm the presence of a single compound for ethyl acetate fractions using HPTLC.

### 2.6. Identification and Quantification of KA Using HPTLC-VIS 

A Camag microliter syringe with a 0.22 µm syringe filter was used to filter the methanol extracts. Next, a Linomat 5 (Camag, Muttenz, Switzerland) was used to apply spots (5 µL) of methanol extracts (5 mg/mL) of *Ocimum basilicum* on a 20 × 10 cm sheet of silica gel 60 Fluorescent 254 (F_254_) (E Merck, Darmstadt, Germany). The solvent solution was formed by toluene, ethyl acetate, and formic acid (70:30:1, *v*/*v*/*v*). The TLC plates were heated at 100 °C for 3 min and dipped into a reagent (2-aminoethyl diphenylborinate) before drying for 2 min in a cool air stream. The plate was subsequently dipped into a polyethylene glycol 400 reagent and allowed to dry for 5 min in a cool air stream. A Camag TLC scanner at 366 nm in absorbance mode was used for the densitometric scanning. A regression equation based on a calibration curve of the KA standard (Sigma Aldrich, St. Louis, MO, USA) was used to quantify KA concentrations.

### 2.7. Calibration Curve Preparation 

KA in HPLC-grade methanol was prepared as a 1000 µg/mL stock solution. Various concentrations (i.e., 0.05, 0.1, 0.2, 0.3, and 0.4 µL) of the stock solution were spotted in order to achieve KA concentrations (50, 100, 200, 300, and 400 ng/spot) on three silica gel plates. 

### 2.8. Response Surface Design

The presence of KA in the methanolic extracts of *Ocimum basilicum* was estimated from 14 fractions of column chromatography. The extraction process was optimized using a Box–Behnken response surface design (BBD) with three factors at three levels. The three factors were methanol concentration (X_1_, %), extraction temperature (X_2_, °C), and extraction time (X_3_, min). The factor levels are shown in Table 2. The extraction yield of KA was the response variable. Design-Expert version 12 software was implemented using the following second-order polynomial model to describe the effect of the factors and levels:

(1) $$Y=b_{0}+\overset{3}{\underset{i=1}{\sum }}b_{i}X_{i}+\overset{3}{\underset{i=1}{\sum }}b_{\mathrm{ii}}X^{2}{}_{i}+\overset{3}{\underset{i\neq j=1}{\sum }}b_{\mathrm{ii}}X_{i}X_{j}$$

where *Y* is the predicted response b_0_ is the intercept; b_1_, b_2_, and b_3_ are the linear coefficients of methanol concentration (X_1_), extraction temperature (X_2_), and extraction time (X_3_), respectively; b_11_, b_22_, and b_33_ are the squared coefficient of methanol concentration, extraction temperature, and extraction time, respectively; and b_12_, b_13_, and b_23_ are the interaction coefficients of methanol concentration, temperature of sonication, and extraction time, respectively. The factor settings were represented as X_i_–X_j_.

### 2.9. Validation Method

Several steps were implemented in order to validate the HPTLC method. The range of compound concentrations was measured for linearity, with results expressed as a correlation coefficient from linear regression. A sample was prepared by adding a known concentration of compounds to a previously quantified sample. This sample was analyzed at three different times on the same day in order to assess intra-day precision and daily for 5 consecutive days in order to quantify inter-day precision. Systemic error was calculated in order to estimate the accuracy of the analytical method. The relative standard deviation of the linearity data and a calibration curve slope were used to calculate the limit of detection (LOD) and the limit of quantitation (LOQ) for KA. 

### 2.10. Free Radical Scavenging Activity

Methanol extracts and KA were evaluated for antioxidant activities using the 2,2-diphenyl-1-picryl-hydrazyl-hydrate (DPPH) radical scavenging method described by Mensor et al. [27], with slight modifications. Dimethyl Sulfoxide DMSO (SIGMA) was used to dissolve test samples and mixed with 20 mg/mL of a DPPH methanol solution in order to yield concentrations of 10, 50, 100, 500, and 1000 μg/mL. After all samples were allowed to sit at room temperature for 30 min, absorbance values were measured at 517 nm and converted into the percentage of antioxidant activity as follows:

% Inhibition = (Absorbance of control − Absorbance of test sample) × 100/Absorbance of control

Butylated hydroxytoluene (BHT) and L-ascorbic acid were used as reference standards with the unit of probits for the inhibition ratio. Probit values were plotted against the logarithmic values of test samples concentrations using a linear regression curve to establish the half maximal inhibitory concentration (IC_50_) values (μg/mL). All analyses were repeated in triplicate, with results expressed as mean ± standard deviation (SD) and compared using the Waller–Duncan test with alpha = 0.05.

### 2.11. Surface Method Model Response and Validity Testing

Experiment results were analyzed with the Design-Expert™ (version 12) software (alpha = 0.05). The three factors of methanol concentration, extraction temperature, and extraction time were simultaneously optimized using the response surface method (RSM). A subsequent ultrasonic-assisted extraction experiment was completed in triplicate using the optimized conditions, and the KA yield was compared with the predicted values for the validation of the model. A mean comparison using Tukey’s test was performed with STATISTICA 13 (alpha = 0.05).

## 3. Results and Discussion

### 3.1. Screening of Total Flavonoid Content in Basil Leaves 

**Figure 1:**
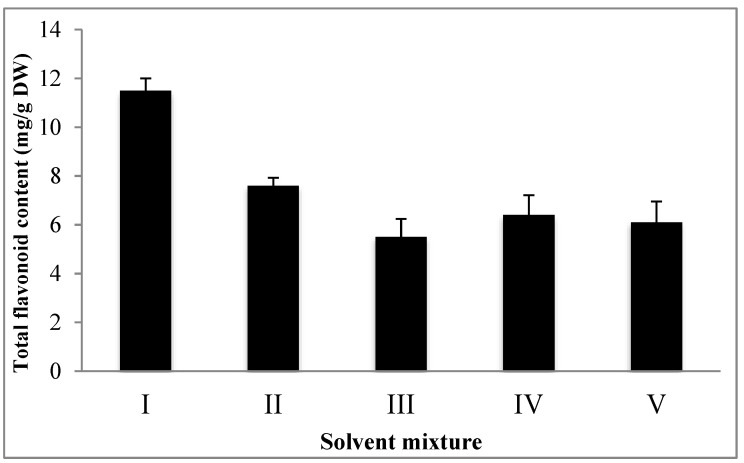
Type of solvent mixture used for extraction.

The total flavonoid content from basil leaves was determined in this study. The solvent mixtures used for the extraction contained different ratios of methanol, water, acetone, and acetic acid (Table 1). The different solvent mixtures used for the extraction significantly (*p* < 0.05) affected the total flavonoid content. The methanol/water (50/50, *v*/*v*) solvent extraction provided the highest yield, whereas the methanol/acetic acid (90/10, *v*/*v*) solvent extraction had the lowest flavonoid content when performing extraction on freeze-dried materials (Figure 1). Solvent polarity played a critical role in the extraction of total flavonoid content, and the methanol/water mixture led to the best performance acting as a great solvent system for polar antioxidants [28,29].

Several studies have been conducted to develop efficient procedures for the large-scale industrial application of natural antioxidants. Due to higher yields, ethanol and methanol are commonly used as solvents to extract flavonoids [30]. However, in a study conducted by Kobus-Cisowska et al. [31] on *Morus alba* fruits, acetone-based mixtures turned out to be more effective than methanol-based mixtures for phenolic and flavonoid extractions. In order to optimize and maximize extraction procedures, it is critical for researchers to take the food matrices and the concentration of the solvent into consideration when extracting phenolic compounds and flavonoids from herbal plants [32]. 

### 3.2. HPTLC-VIS Analysis of KA

**Figure 2:**
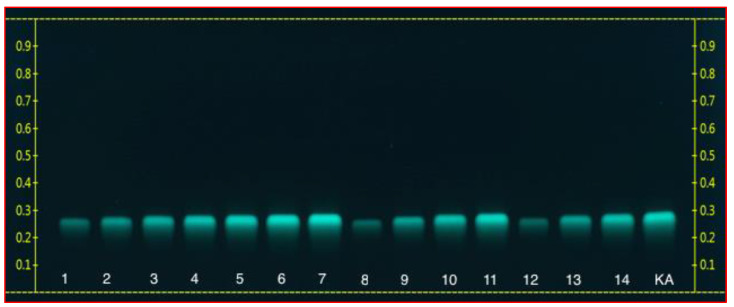
Photograph of thin layer chromatography (TLC) of ethyl acetate fraction at 366 nm. Kaempferol (KA); spots: 1–14 for ultrasonicated *Ocimum basilicum* extracts.

**Figure 3:**
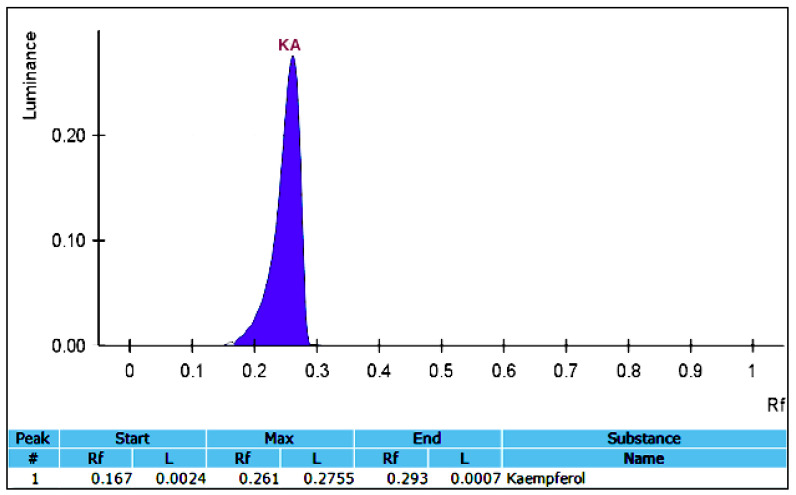
High performance thin layer chromatography (HPTLC) chromatogram of the KA standard. Rf, Retention factor. #, number; L, lowest distance.

**Figure 4:**
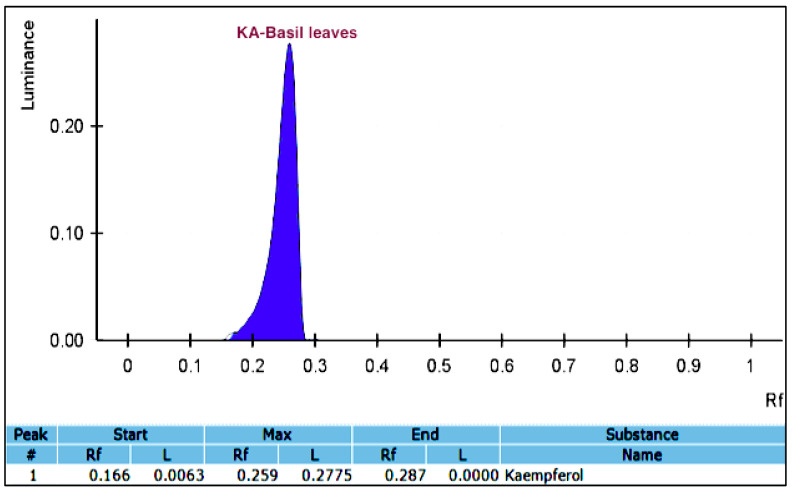
HPTLC chromatogram of KA in the methanol extract of ultrasonicated basil leaves (*Ocimum basilicum*) under the optimized conditions. Rf, Retention factor. #, number; L, lowest distance.

From the screening study, it was shown that the methanol–water solvent exhibited the highest yield of flavonoids from basil leaves when compared to the other four solvents. As a consequence, this type of extraction solvent was selected for the KA extraction. The ultrasonication extraction technique was performed on the extraction of the KA-containing methanol extract. To separate the bioactive compounds, the ultrasonicated *Ocimum basilicum* extracts from 15 experiments were partially purified with sequential purification through column chromatography. The results obtained from the column chromatography showed that among the hexane, ethyl acetate, and petroleum ether extractions, only ethyl acetate impacted the presence of KA in the extract. The ethyl acetate extraction was subsequently selected for TLC analysis. The test tubes were collected and the mobile phase was evaporated in order to obtain the pure, yellow crystals from the ethyl acetate fractions. The KA band in the ultrasonicated *Ocimum basilicum* extracts was verified by being compared with a standard of the KA sample using TLC (Figure 2). A validated HPTLC-VIS technique was used to quantify the KA for all of the experimental Box–Behnken design (BBD) runs. A solvent system consisting of toluene-ethyl acetate-formic acid (70:30:1 *v*/*v*/*v*) was selected, and this system provided a sharp and well-defined KA peak at an Retention factor (Rf) value of 0.26 (Figure 3). To our knowledge, this is the first report of the solvent system used for the isolation of KA. The clear resolution exhibited on the chromatography demonstrates that this solvent system is capable of efficiently separating KA from the methanol extract of the ultrasonicated *Ocimum basilicum* (Figure 4). 

Sample concentrations within the range of 50–400 ng/spot were selected to study the range of linearity (Table 3). The obtained data were close to unity and fit a linear model (R^2^ = 0.99). Validation parameters such as the LOD and the LOQ were used to confirm the accuracy and precision of the proposed method with reproducible results. The validation parameters in the current study were large (LOD = 8.16 ng; LOQ = 18.142 ng), indicating that the method used here was more sensitive than the HPTLC methods used for previous KA analyses [33]. A recovery exceeding 98% was observed for the accuracy study (Table 4). For the precision test, a recovery of less than 2% was observed for the relative standard deviation (RSD) for inter-day and inter-day variations, suggesting that the proposed method had outstanding precision (Table 5). 

### 3.3. Model Fitting

HPTLC-VIS was used to quantify the KA yield from each experimental BBD run (Table 6). A regression analysis was performed, and four proposed models were analyzed. The quadratic model appeared to be the best fit (adjusted R^2^ = 0.9138), followed by the cubic model (adjusted R^2^ = 0.9033), 2 Factor Interaction (FI) model (adjusted R^2^ = 0.5462), and linear model (adjusted R^2^ = 0.1986) (Table 7). The adjusted R^2^ of the quadratic model was close to 1, implying a certain degree of correlation between the observed and predicted values. The adequate precision was calculated in order to determine the signal-to-noise ratio, and in this study, the adequate precision reading was 17.58, which was larger than the desired value (>4.0). Such a large value would be indicative of an adequate signal and could be used to navigate through the design space. The analysis of variance (ANOVA) of the fitted quadratic polynomial model for the KA yield is presented in Table 8. The lack of a fit F-value test for the model described the deviation in the data around the fitted model. The obtained value was not significant (*p*-value = 0.265), indicating that the result might have been due to pure error, which therefore validated the RSM results. In other words, if the model did not adequately fit the data, the resultant lack-of-fit value would be significant. The optimization of a fitted response surface is likely to provide false results [34]. The model in this study was significant (*p*-value = 0.082). The achieved results were in agreement with previous published data on *Ocimum sanctum* where an HPTLC method was developed involving a Box–Behnken-supported design for the simultaneous optimization, validation, and quantification of polyphenols in an aqueous alcoholic extract [35]. 

### 3.4. Effect of Extraction Parameters on KA Yield of Ultrasonicated Ocimum Basilicum and RSM Analysis

The ANOVA and the contributions of each independent variable for the extraction of KA from ultrasonicated *Ocimum basilicum* were determined. The linear, interaction, and quadratic variables were significant (*p* < 0.05), thus indicating that the KA yield was affected by all of the variables, the interactions between variables, and the square of each variable. The model in the study showed a high degree of precision (R^2^ = 0.9735), and the experiment values were reliable (% Coefficient of Variation (CV) = 0.55) [36]. Aiming to visualize the relationship between the independent variables and the KA yield, three-dimensional (3D) plots were constructed based on the generated quadratic polynomial model equation of coded factors:

KA yield = 90.15 + 0.4375 × A + 1.2875 × B + 1.73543 × 10^−15^ × C + 1.75 × AB + 0.025 × AC + −0.725 × BC + 1.2 × A^2^ + 1.89196 × 10^−14^ × B^2^ + −0.825 × C^2^

The parameter variables in this study were methanol concentration (A), extraction temperature (B), and extraction time (C). The effects of the parameter variables and their interactions on the KA contents in the ultrasonicated *Ocimum basilicum* were examined. The third variable was assigned to be constant at the intermediate setting, while the two independent variables were displayed on three-dimensional surface plots. 

**Figure 5:**
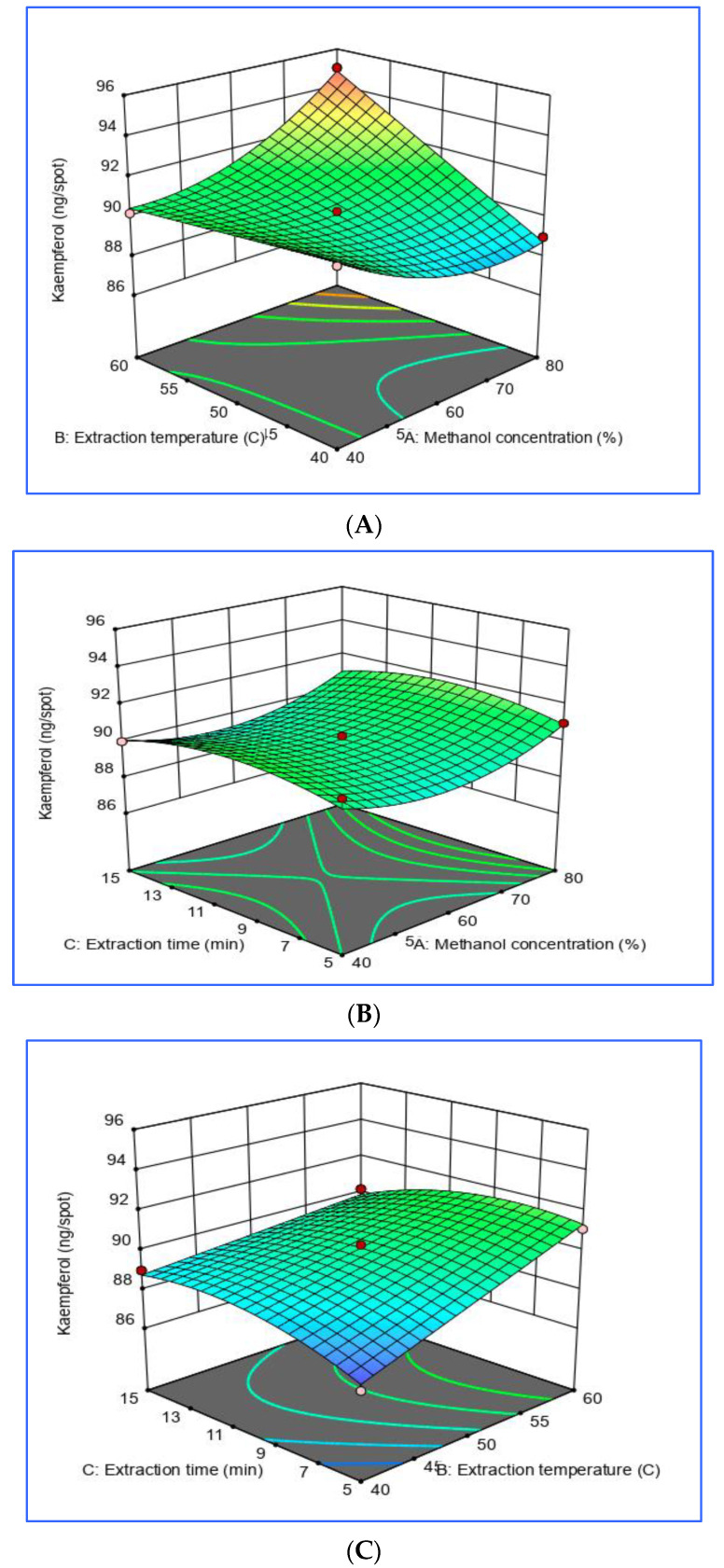
Response surface model 3D plots showing the effects of independent variables of ultrasonicated *Ocimum basilicum* on KA yield. (**A**) Methanol concentration and extraction temperature, (**B**) methanol concentration and extraction time, and (**C**) extraction temperature and extraction time.

Figure 5A shows that the yield of the KA content rapidly increased and reached the maximum value at 0 level of extraction time when the methanol concentration increased from 40% to 79.99%. The extraction temperature was fixed at 60.02 °C. Figure 5B shows how the KA content was affected by the interaction between the methanol concentration and the extraction time at a fixed extraction temperature level of 0. The maximum KA content was obtained when the methanol concentration was at 80%. The KA yield content decreased slightly when the methanol concentration was increased to 80%. The extraction time was fixed at 5.0 min. Figure 5C shows that the KA yield rapidly increased and reached the maximum value at 0 level of methanol concentration when the extraction temperature increased from 40 to 60 °C. The extraction time was fixed at 5.0 min. Interestingly, when the extraction temperature exceeded 60 °C, the yield of the extracted KA rapidly decreased. This result confirmed the fact that the solubility of the solute was enhanced when the temperature increased, resulting in a higher yield. However, the solvent density could be simultaneously reduced when the temperature increased, consequently decreasing the total flavonoid yield. This would mean that either a positive or negative effect could occur when the temperature increased [37]. This finding is in agreement with the study conducted by Peng et al. [38] in which the thermal degradation of flavonoids and the yield of KA decreased when the number of acoustic cavitation bubbles decreased. 

### 3.5. Optimization and Verification of the Model for Extraction Parameters

Design-Expert^TM^ software (version 12) was used to determine the optimized extraction process parameter. The desirability function was applied to the optimizing stage in order to maximize the yield of KA. The optimal conditions for the three parameters of the extraction process were estimated: The methanol concentration was 79.99%, the extraction temperature was 60.02 °C, the extraction time was 5.0 min, and the KA yield was 94.7 ng/spot. The extraction process was repeated, the optimum extraction conditions, i.e., an 80% methanol concentration at 60 °C for 5 min, and the KA yield was 94.65 ± 0.5. There was no significant difference (*p* > 0.05) between the predicted value and the experiment value, which demonstrated that the model proposed in this study can be utilized for extracting KA from ultrasonicated *Ocimum basilicum.*

### 3.6. RSM Validation

**Figure 6:**
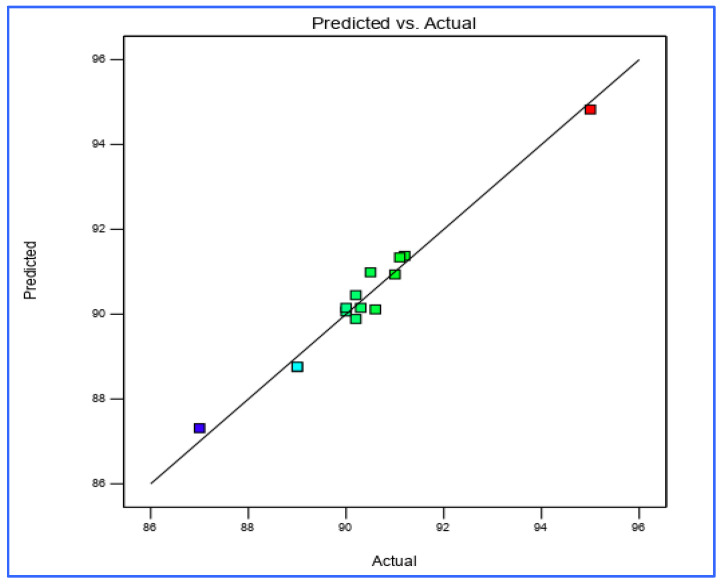
Linear correlation plot between actual and predicted values for KA yield.

The KA yield evaluation results were found to be within the limits of the yield checkpoint. Further validation was performed on the RSM results, and the percentage prediction error was found to be 0.789% when comparing the experiment values of the response with the anticipated values. This provided sufficient evidence to establish the validity of the generated equation and to describe the domain of applicability of the RSM model. The goodness of fit was excellent for the predicted and experiment value linear correlation plot (R^2^ = 0.9905; *p* < 0.05) (Figure 6). Overall, there was a high prognostic ability due to the low magnitudes of error and the significant values of R^2^. 

### 3.7. Antioxidant Activity

Data on the antioxidant activities of the methanol extract of ultrasonicated basil leaves (*Ocimum basilicum*) under the optimized conditions, as well as their isolated compound (KA), are presented in Table 9. The antioxidant activity of the methanol extract of ultrasonicated *Ocimum basilicum* was IC_50_ = 50.10 μg/mL. In contrast, the antioxidant activity of KA (IC_50_ = 0.68 μg/mL) was higher with respect to the reference ascorbic acid (IC_50_ = 0.79 μg/mL) and BHT (IC_50_ = 0.88 μg/mL). The present study was in accordance with the work of Shafique et al. [39], who reported that *O. basilicum* extracts exhibited and offered higher antioxidant activity compared to synthetic antioxidant BHT. 

## 4. Conclusions

The data obtained from the current study showed that the HPTLC-VIS densitometric method and the BBD were efficient for the identification and quantitative analysis of KA from basil leaves. The interaction and the quadratic terms of each factor from all three variables had significant effects on the KA yield. A quadratic model for the KA yield was derived with R^2^ = 0.99. The current study revealed that the use of methanol (79.99%) at 60 °C for 5 min comprised the optimal conditions for extracting a high yield of KA from ultrasonicated basil leaf extracts. Under these conditions, the KA yield was 94.7 (ng/spot), which was consistent with the predicted yield value. The isolated KA exhibited and offered a higher antioxidant activity compared to the reference of ascorbic acid and BHT. This study thus demonstrated, for the first time, the utilization of a solvent system that can be used with higher efficiency during the HPTLC-VIS analysis of KA. The used solvent system provided a high quality resolution of KA peaks. The LOD and LOQ values were found to be comparatively low, thus supporting the high sensitivity of the developed method. In addition, this method was supported by statistical analysis that highlighted how the proposed method is reproducible and specific for this type of extraction.

## Figures and Tables

**Table 1:** Solvent mixtures tested to maximize extraction of total flavonoids.

Solvent Mixture Code	Composition of Solvent Mixture
I	Methanol/water (50/50, *v*/*v*)
II	Acetic acid/acetone/water (10/60/30)
III	Methanol/acetic acid (90/10, *v*/*v*)
IV	Methanol/water/acetone (40/40/20, *v*/*v*/*v*)
V	Absolute methanol

**Table 2:** Extraction variables selected for *Ocimum basilicum* optimization. KA: kaempferol.

Independent Variable	Ranges of Independent Variable			Dependent Variable	Goal
	−1	0	+1		
Methanol concentration (%)	40	60	80	KA Yield	Maximized
Extraction temperature (°C)	40	50	60		
Extraction time (min)	5	10	15		

**Table 3:** Validation parameters for High-Performance Liquid Chromatography (HPLC). LOD: limit of detection; LOQ: limit of quantitation. Rf, Retention factor.

Validation Parameter	Value
Linearity range	(50–400) ng/spot
Correlation coefficient	0.99
LOD (ng)	8.16
LOQ (ng)	18.142
Specificity	Specific
Rf value	0.261

**Table 4:** Data of accuracy for KA.

Concentration (ng/spot)		Amount of KA Found (Mean)	SD	%RSD	%Recovery (*n* = 3)
Taken	Added				
150	0	148.90	0.59	0.401	99.26
150	25	171.88	0.71	0.413	98.21
150	50	197	0.57	0.289	98.50
150	75	222	0.66	0.297	98.66
150	100	247	0.43	0.174	98.80

SD = standard deviation; RSD = relative standard deviation.

**Table 5:** Data for inter-day and intra-day precision for KA.

Concentration (μg/spot)	Inter-Day Precision (*n* = 5)			Intra-Day Precision (*n* = 3)		
	Peak Area (Mean)	SD	%RSD	Peak Area (Mean)	SD	%RSD
5	875	9.77	1.11	922.11	1.14	0.12
10	1172.23	9.98	0.85	1290.88	0.94	0.07
15	1388.16	1.49	0.10	1465	7.77	0.53
20	1642.99	1.13	0.06	1805.29	3.31	0.18
25	1923.23	1.09	0.05	2218.22	3.03	0.13

SD = standard deviation; RSD = relative standard deviation.

**Table 6:** Box–Behnken design (BBD) matrix for the optimization of extraction of KA yield (ng/spot).

Run	Methanol Concentration (A) (%)	Extraction Temperature (B) (°C)	Extraction Time (C) (min)	KA (ng/spot)
1	60	50	10	90
2	80	60	10	95
3	80	50	5	91
4	60	60	5	91.1
5	40	40	10	91.2
6	40	60	10	90.2
7	40	50	15	90
8	80	40	10	89
9	60	50	10	90.3
10	40	50	5	90.6
11	60	60	15	90.2
12	60	40	15	89
13	80	50	15	90.5
14	60	40	5	87

**Table 7:** Regression analysis results.

**KA Yield**	**Model F Value**	**R^2^**	**Adjusted R^2^**	**Predicted R^2^**
	Linear	0.3835	0.1986	−0.3353
	2FI	0.7557	0.5462	−0.3151
	Cubic	0.8924	0.9033	-
	Quadratic	0.9735	0.9138	0.9088

FI (factor interaction).

**Table 8:** Analysis of variance (ANOVA) results for KA extraction.

Source	Sum of Square	Degree of Freedom	Mean Square F	F-Value	Prob > F
Model	37.55	9	4.17	16.32	0.0082
Residual	1.02	4	0.2556	-	-
Lack of fit	0.9775	3	0.3258	7.24	0.2651
Pure error	0.0450	1	0.0450	-	-

**Table 9:** Inhibition concentrations of test samples scavenging 50% of the 2,2-diphenyl-1-picryl-hydrazyl-hydrate (DPPH) radical.

Test Samples	IC_50_ (μg/mL)
Methanol extract of ultrasonicated *Ocimum basilicum*	50.10 ± 0.513
Isolated KA	0.68 ± 0.021 ^c^
L-ascorbic acid	0.79 ± 0.015 ^b^
BHT	0.88 ± 0.026 ^a^

Half maximal inhibitory concentration (IC_50_). ^a^, ^b^, ^c^ Means with different superscripts are significantly different at *p* ≤ 0.05.
